# Structural basis of a novel heterodimeric Fc for bispecific antibody production

**DOI:** 10.18632/oncotarget.17558

**Published:** 2017-05-02

**Authors:** Hudie Wei, Haiyan Cai, Yuhao Jin, Pilin Wang, Qingqing Zhang, Yihui Lin, Weixiao Wang, Jinke Cheng, Naiyan Zeng, Ting Xu, Aiwu Zhou

**Affiliations:** ^1^ Hongqiao International Institute of Medicine, Shanghai Tongren Hospital/Faculty of Basic Medicine, Key Laboratory of Cell Differentiation and Apoptosis of The Chinese Ministry of Education, Shanghai Jiao Tong University School of Medicine, Shanghai, China; ^2^ The Therapeutic Antibody Research Center of SEU-Alphamab, Southeast University, Nanjing, China; ^3^ Division of Translational Medicine, 3D Medicines Corporation, Shanghai, China; ^4^ Department of Pharmaceutical Engineering, College of Humanities-Information, Changchun University of Technology, Changchun, China; ^5^ Department of Biochemistry and Molecular Cell Biology, Shanghai Key Laboratory for Tumor Microenvironment and Inflammation, Institute of Medical Sciences, Shanghai Jiao Tong University School of Medicine, Shanghai, China

**Keywords:** bispecific antibodies, heterodimeric Fc engineering, crystal structure, trastuzumab, pertuzumab

## Abstract

Bispecific antibodies provide an efficient tool for combinational clinical therapy. Here we have engineered a heterodimeric Fc for bispecific antibodies production by combining the knob-into-hole and electrostatic steering strategies where a bulky hydrophobic residue Phe405 of the IgG CH3 interface is mutated to a charged residue Lys and Lys409 of the corresponding CH3 domain is mutated to Ala. The crystal structure of this Fc heterodimer solved here at 2.7Å resolution revealed how these two mutations resulted a complementary binding interface and explained why F405K mutation could effectively inhibit Fc homodimer formation during protein expression. An anti-HER2 bispecific antibody derived from trastuzumab and pertuzumab was generated by this heterodimeric Fc. It showed comparable or improved efficacy than the combination of trastuzumab and pertuzumab in inhibiting proliferation of cancer cells *in vitro* and *in vivo*. Overall this study shows that the heterodimeric Fc engineered here provides an efficient platform for generating active bispecific antibody for cancer treatment.

## INTRODUCTION

Antibody represents a major class of biopharmaceuticals in effective treatment of tumor, inflammatory, infectious and many other diseases [1]. About 50 monoclonal antibody (mAb) products have been approved for therapy in the US and Europe, with more than 400 in clinical trials [2]. Several antibodies against human epidermal growth factor receptor 2 (HER2, ErbB2) such as trastuzumab and pertuzumab are in clinical use for HER2-positive solid tumor. HER2 is a member of human epidermal growth factor receptor family, including HER1(EGFR), HER2, HER3 and HER4 [3]. Each receptor is constituted by three domains: an intracellular protein kinase domain, an alpha helical transmembrane segment, and an extracellular domain which is subdivided into four further domains [4]. HER2 has no direct ligand and may be activated by heterodimerization with other family members, thereby initiating downstream MAPK and PI3K signaling pathways [5, 6]. It plays an important role in proliferation, differentiation and survival of normal and cancer cells [7]. Overexpression of HER2 is found in 25%–30% of breast cancers [8, 9], 17% to 22% of gastric cancers [10, 11], 4% to 6% of non–small cell lung cancers (NSCLC) [12, 13] and also many other kinds of cancers. Trastuzumab is a humanized mAb that binds extracellular domain IV of HER2. It has made potent improvements in survival of metastatic breast cancer [14–17]. Pertuzumab, a newer humanized anti-HER2 antibody, has a different mode of action with trastuzumab. It directly binds to the extracellular dimerization domain (subdomain II) of HER2 and prevents dimerization of HER2 with other ErbB family proteins [18–20].

Since complex diseases, including cancer, are often multifactorial, mAbs with a defined specificity may not exert sufficient desired effect [21]. Many cancer patients do not respond to trastuzumab initially or get acquired resistance within one-year treatment [22, 23]. The proposed mechanism of resistance to trastuzumab involves heterodimerization between HER2 and other ErbB members, whereas trastuzumab seems to be insufficient in blocking HER2 heterodimerization [19, 23–25]. Nevertheless it has been reported that combination of trastuzumab and pertuzumab provides a more efficient blockade of HER2 signaling than either antibody alone, leading to a more effective inhibition of tumor growth in HER2-positive breast cancer, gastric cancer and lung cancer [26–28]. Pertuzumab plus trastuzumab plus docetaxel was approved for the first-line treatment of patients with HER2-positive metastatic breast cancer, and an phase III study exhibited a effective results for the first-line pertuzumab, trastuzumab and chemotherapy in HER2-positive metastatic gastric and gastro-oesophageal junction cancer [29–31].

Therefore bispecific antibody (bsAb), which can simultaneously recognize two different antigens or two distinct epitopes on the same antigen would have advantages for clinical application in cancers and immune disorders [32–35]. A variety of bsAb formats have been reported, including mAb catumaxomab marketed in Europe which is a mouse/rat hybrid immunoglobulin G (IgG) [2], tandem single chain variable fragments (scFv) represented by Blinatumomab, diabodies, IgG-like dual variable domain antibodies, and IgG-scFv antibodies [1, 2]. However, most bsAb formats encounter difficulties in large-scale manufacturing due to poor physicochemical properties. Meanwhile, bsAb formats without Fc portion usually has much shorter *in vivo* half-life [1, 36, 37]. To minimize these problems, we tried to build our bsAbs based on heterodimeric Fc technologies. The assembly of light chains can be solved with a common light chain [38–40] or with the cognate light chain by CrossMab technology [41]. To favor heterodimerization over homodimerization of Fc, the knob-into-hole (KiH) technology was developed previously based on the crystal structure of human IgG1 Fc [42–44]. “Knob” means replacing a small amino acid with a larger one in one CH3 domain (such as T366W) [43] and accordingly replacing a large amino acid with a smaller one as “hole” in the other interactional CH3 domain (such as T366S/L368A/Y407V) [44]. KiH Fc variants thermodynamically favor the formation of heterodimers rather than homodimers. However, the yield of heterodimer was still somewhat unsatisfying even after extensive optimization by phage display. Other heterodimeric Fc variants, such as ZW1 [45], EW-RVT [46, 47], HA-TF, SEEDbodies [48], DuetMab [49], electrostatic steering [50], have also been developed.

To further improve the KiH platform, here we mutated a bulky hydrophobic residue Phe405 of the IgG CH3-CH3 interface to a charged residue Lys and Lys409 of the corresponding CH3 domain to Ala. The mutations strongly favor the formation of heterodimer while prevent homodimeric interactions. The crystal structure of this Fc heterodimer solved here reveals the detailed interactions. For practical use, an anti-HER2 bsAb KN026 was generated using this heterodimeric Fc and showed comparable or even better efficacy than the combination of trastuzumab and pertuzumab *in vitro* and *in vivo* experiments. Currently, all Investigational New Drug (IND) enabling work for KN026 has been completed with first-in-man study and IND application will be initiated later in 2017.

## RESULTS

### Design and evaluation of the heterodimeric Fc variant

**Figure 1:**
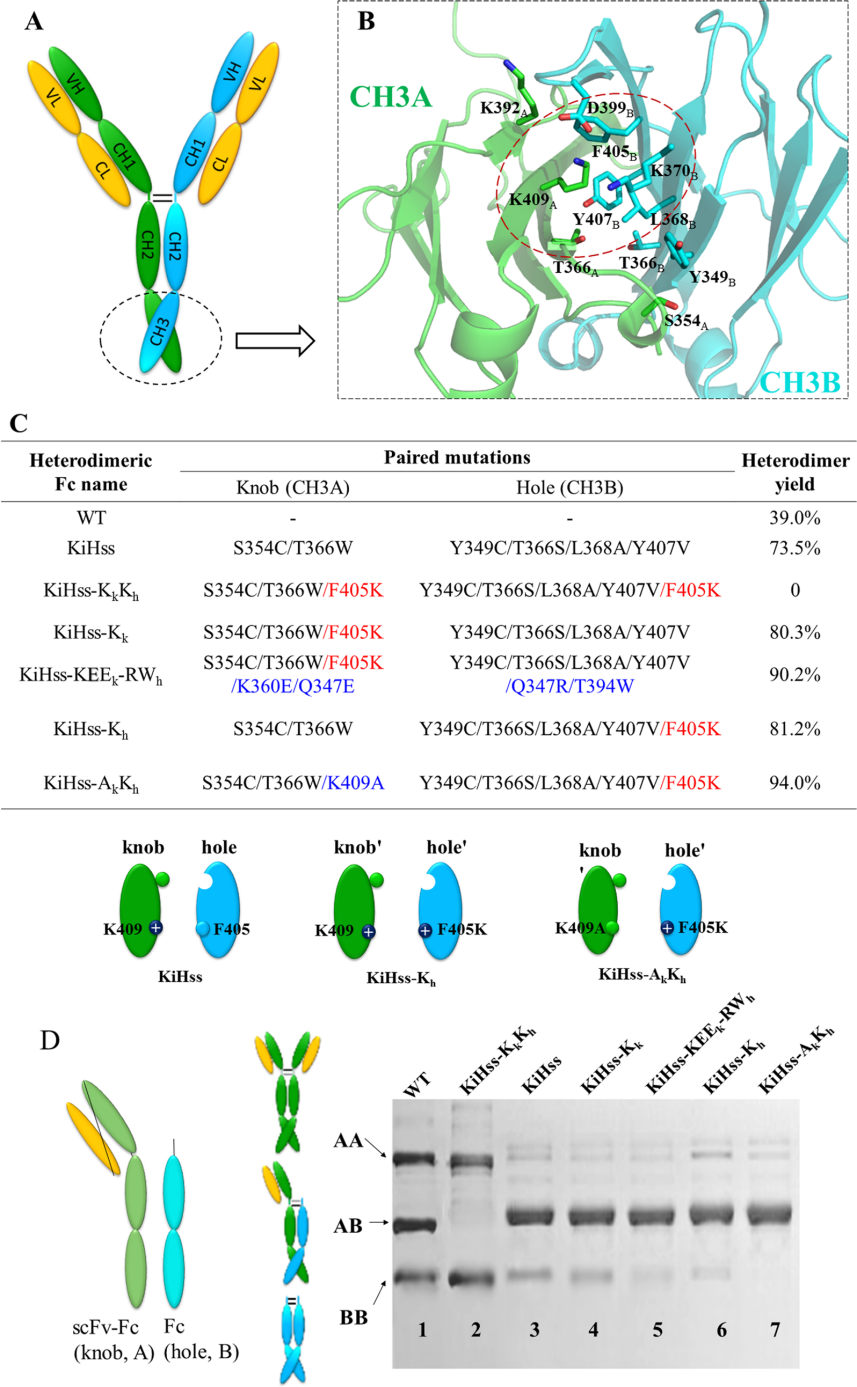
Design of Fc heterodimer (**A**) A schematic of human IgG1. (**B**) The structure of CH3 domains of wild-type Fc (PDB: 3AVE). Key residues involved in CH3-CH3 interface are showed as sticks. Green, CH3A; cyan, CH3B. (**C**) Fc heterodimer engineering based on the knob-hole strategy (KiH). (**D**) Knob (A) and hole (B) chain formation was analyzed by non-reducing SDS-PAGE. The yield of Fc heterodimer was assessed by densitometry analysis and shown in C.

Non-covalent CH3-CH3 interactions form the basis of Fc homodimerization and are subsequently enhanced by covalent disulfide linkages in the hinge region (Figure 1A). Structures of human IgG1 Fc show that Fc homodimerization is driven by both hydrophobic interactions at the center of the CH3 interface core and symmetric electrostatic interactions surrounding the rim of the hydrophobic core [42, 51]. Although the knob-into-hole Fc technology has made huge progress in designing bsAb with engineered asymmetric CH3 domains. Previous reports showed that knob mutation such as T366W is effective in disfavoring knob chain homodimer formation due to the steric hindrance of its bulky side chain [39, 43, 44, 52], however it is not so effective in preventing homodimerization of the hole chain. Here we have closely evaluated the interface between the CH3 domains of Fc and that of knob-into-hole Fc variant (knob-T366W, hole-T366S/L368A/Y407V, PDB: 4NQS) (Figure 1B, Supplementary Figure 1) and found that F405 in a hydrophobic patch forms close contact with K409 of the other CH3 domain (Figure 1C). Notably the electrostatic interactions of K409-D399 pair in the rim of CH3 interface contributes to Fc association. Mutations of this pair by charge reversal strategy (CH3A-K409D/K392D, CH3B-D399K/E356K) or by engineering complementary hydrophobic interactions (CH3A-K409W, CH3B-D399V/F405T) would suppress Fc homodimer formation [50, 53]. Therefore we predicted that replacement of F405 in chain B with a positively charged residue Lys would reduce its homodimer formation. Accordingly mutation of K409 in chain A to an Ala would eliminate potential clash with residue F405K in chain B during heterodimer formation (Figure 1C). At the same time, we also introduced a disulphide bond between the CH3 chains by generating S354C and Y349C mutation which have been reported to efficiently improve the heterodimer yield [39].

The effect of these mutations on the heterodimeric Fc formation was assessed by the scFv-Fc/Fc system where a Fc chain (Hinge-CH_2_-hole CH3B, B) and a scFv-Fc chain (VH-linker-VL-Hinge-CH_2_-knob CH3A, A) were co-transfected with a ratio of 1:1 and expressed in HEK293 cells (Figure 1D). The secreted Fcs in the expression medium were purified by protein A affinity chromatography, then analyzed by non-reducing SDS-PAGE. Once assembled, the two chains of Fc will be linked by two disulphide bridges in the link region. As shown in Figure 1D with the wild-type Fc template, about 40% of total Fcs form heterodimeric Fc (AB) and there was slight more dimers of scFv-Fc (AA) than those of Fc (BB) (Lane 1). KiH mutations plus engineered disulphide bond (mutant KiH_ss_) significantly improved the yield of heterodimeric Fc to about 73% (lane 3). Introducing F405K mutation into knob chain (mutant KiH_ss_-K_k_, lane 4) or hole chain (mutant KiH_ss_-K_h_, lane 6) increased the yield of heterodimer to more than 80%. Further introduction of ionic interactions as previously described around the rims Fc interface onto KiH_ss_-K_k_ mutant gave a yield of ~90% (lane 5). The most significant formation of heterodimeric Fc was obtained from the combinations of mutation K409A in knob chain and F405K mutation in the hole chain (mutant KiH_ss_-A_k_K_h_) with 94% of Fc in heterodimeric form (lane 7). Also this combination seemed to have abolished the Fc homodimer formation of the hole chain (lane 7). Most remarkably, when F405K mutation was introduced into both knob and hole chain (mutant KiH_ss_-K_k_K_h_), it completely blocked the formation of heterodimer (lane 2). These data indicate F405K mutation plays a critical role in preventing the Fc homodimer formation leading to higher yield of Fc heterodimers.

### Structure basis of heterodimer formation

**Figure 2:**
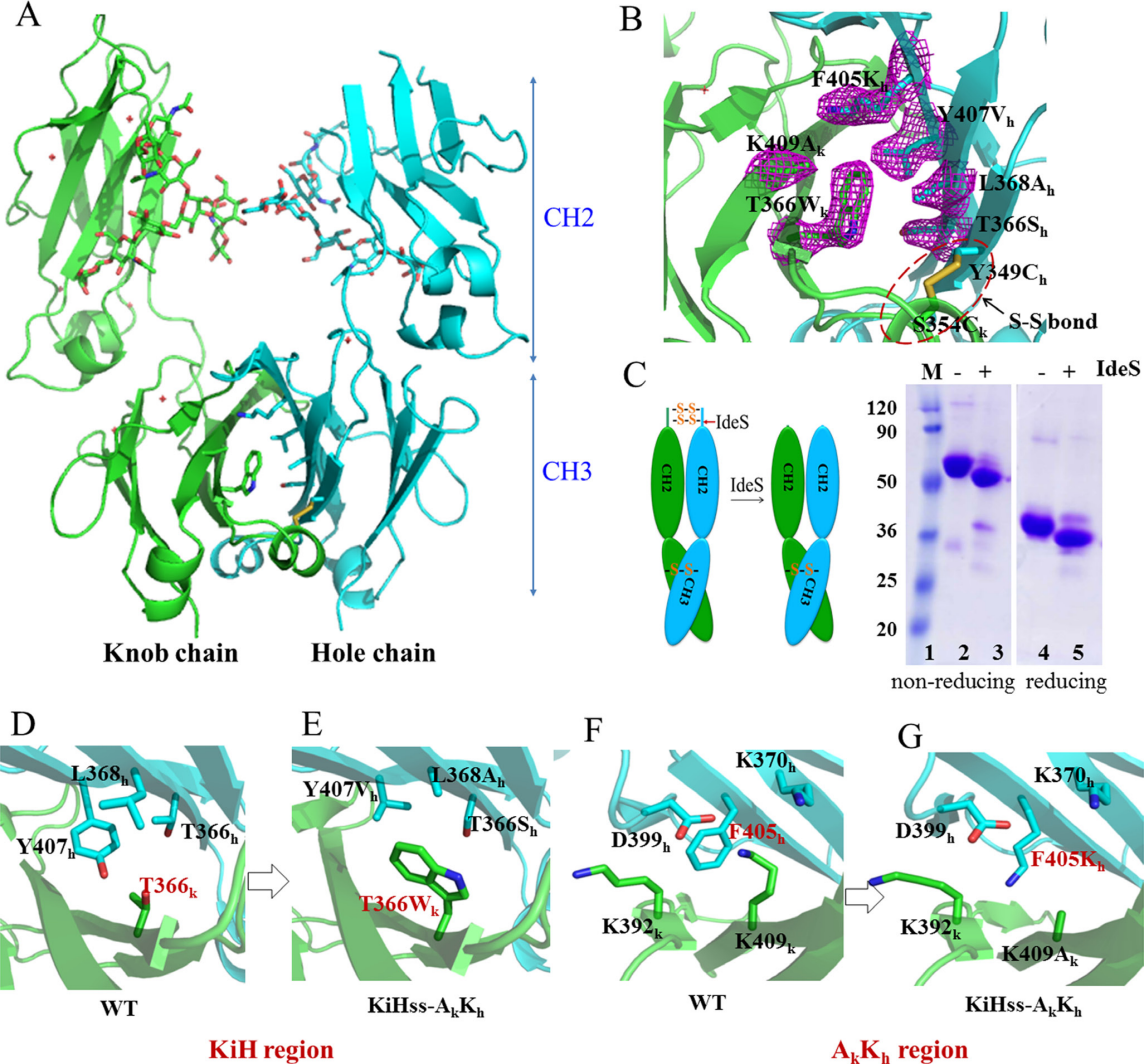
Structural basis of Fc heterodimer formation (**A**) Overall structure of Fc variant KiH_ss_-A_k_K_h_. Carbohydrates are shown as sticks. Green, knob chain; cyan, hole chain. (**B**) The CH3-CH3 interface of KiH_ss_-A_k_K_h_. The mutated residues are showed as sticks, with electron density map contoured at 1σ. (**C**) Process of Fc variant KiH_ss_-A_k_K_h_ by streptococcal IgG endopeptidase (IdeS) indicating the engineered disulphide linkage is properly formed as designed. (**D**, **E**) Interaction of the residues in the knob-into-hole region. In the structure of wild type, the residue T366 of CH3A chain contacts with T366, L368, Y407 of CH3B chain by forming hydrophobic interaction (D), while in the structure of KiH_ss_-A_k_K_h_, the knob T366W_k_ interacts the hole composed of T366S_h_/L368A_h_/Y407V_h_ with optimal hydrophobic interaction, strengthening the heterodimer interactions (E). (**F**, **G**) Interaction of the residues in A_k_K_h_ region. In the structure of wild type, residue F405 is adjacent to charged residues (F) while in the structure of KiH_ss_-A_k_K_h_, the mutation F405K_h_ and K409A_k_ do not influence heterodimer formation (G).

To better understand how F405K mutation favors Fc heterodimer formation, we solved the structure of KiH_ss_-A_k_K_h_ Fc heterodimer (knob-S354C/T366W/K409A, hole-Y349C/T366S/L368A/Y407V/F405K) at 2.7Å resolution with a space group of P 21 21 21 (Table 1). There is one dimer of CH2-CH3 domains in the asymmetric unit (Figure 2A). The overall structure is very similar to that of wild type human IgG with the root-mean-square deviations (RMSD) for superposition of Cα atoms of 0.44Å. Carbohydrates attached to the glycosylation site of the Asn297 residue of both CH2 domains are showing normal glycosylation pattern [54]. Non-covalent interactions between two CH3 domains contribute to heterodimer formation without significant structural perturbations to Fc surface (Figure 2A, 2B). All mutated residues are largely buried in the CH3-CH3 interface (Figure 2B). However the mutant is asymmetry with the RMSD of Cα atoms of the knob and hole chain at 1.02Å. The difference between these two chains is likely induced by the introduced mutations when two chains form complementary complex.

**Table 1:** Data collection and refinement statistics

***Data collection***	
Beamline	SSRF BL 17U1
Space group	P 21 21 21
Cell dimensions	
a, b, c (Å)	49.26, 79.52, 137.98
α, β, γ (°)	90, 90, 90
Wavelength (Å)	0.97915
Resolution (Å)	49.26–2.70 (2.83–2.70)
Total NO. of observation	90719 (11095)
NO. of unique	15314 (1948)
Rmerge (%)	0.114 (0.58)
I/σI	8.6 (2.6)
Completeness (%)	98.6 (96.5)
Multiplicity	5.9 (5.7)
***Model***	
No of atoms	3386
protein	3182
water	8
carbohydrate	196
Average B-factors (Å^2^)	68.8
***Refinement***	
Resolution (Å)	46.39–2.70 (2.77–2.70)
No. of reflections	14450
R_work_/R_free_	0.24/0.29
RMSD	
Bond lengths (Å)	0.007
Bond angles (°)	1.285
Ramachandran plot (%)	97.7/2.3/0^a^
PDB code	5TPS

^a^Preferred region/allowed region/disallowed region

A detailed comparison of structure of KiH_ss_-A_k_K_h_ with that of wild-type Fc or those of other Fc heterodimers indicates that the knob mutation T366W_k_ in our structure is accommodated in a hydrophobic pocket formed by hole mutations T366S_h_/L368A_h_/Y407V_h_. These optimal hydrophobic interactions likely play a key role in stabilizing the heterodimeric interactions (Figure 2D, 2E), which largely resemble these interactions in the previous knob-into-hole variant (PDB: 4NQS). The F405K and K409A mutations designed here form a complementary surface with no perturbations to the nearby residues and the long side chain of Lys occupies the space vacated by those of F405 and K409 (Figure 2F, 2G). To better understand the effect of F405K_h_ on hole-hole homodimerization, a model of hole-hole homodimer was generated by simply replacing the knob chain in the KiH_ss_-A_k_K_h_ dimer with the same hole chain (Supplementary Figure 2). This shows that the sidechains of K405/K370 from one hole chain will be close to residues K392_’_/K409 of the other hole chain. Therefore the electric charge repulsion effect between these residues likely plays a key role in disfavoring hole-hole homodimerization.

The distance between the two α-carbons of S354C_k_ and Y349C_h_ is 5.92Å, which is compatible for a disulphide bond formation. However, due to poor electron density in the side chains of these residues in the structure of KiH_ss_-A_k_K_h_, it is unclear if the designed disulfide bond is correctly formed as designed (Figure 2B). We then assessed the formation of this disulphide bond by limited protease digestion using streptococcal IgG endopeptidase IdeS (Figure 2C). This enzyme specifically cleaves the IgG between the hinge region and Fc of the heavy chains. Under reducing conditions, the Fc fragment containing CH2-CH3 domains migrated as a band around ~36KDa before IdeS treatment and migrates as 33KDa band after IdeS cleavage indicating that IdeS cleaves this Fc fragment as expected. However, under non-reducing conditions, the Fc fragment containing CH2-CH3 domains migrated as a band around ~70KDa before IdeS treatment and migrates as ~60KDa band after IdeS cleavage (Figure 2C). When an antibody with wild-type Fc was treated by IdeS and analysed by SDS-PAGE under non-reducing condition, only a band of ~33KDa corresponding to Fc fragment appeared (Supplementary Figure 3). This indicates that the Fc in KiH_ss_-A_k_K_h_ is still linked by a disulphide bridge after cleavage and the inter-chain disulfide bond between S354C_k_ and Y349C_h_ is formed as expected.

### Construction and characterization of anti-HER2 bsAb KN026

**Figure 3:**
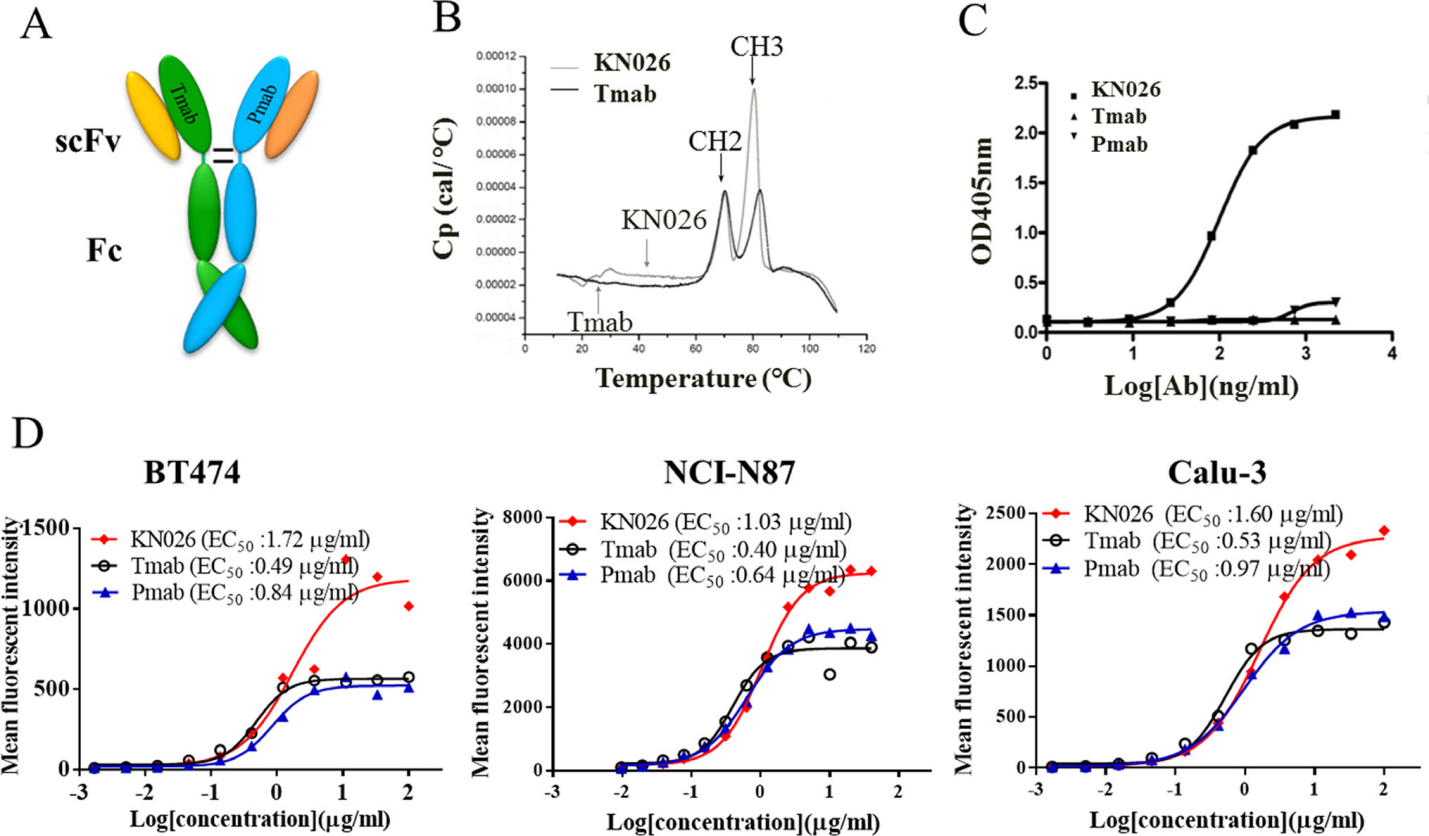
Construction and characterization of anti-HER2 bsAb KN026 (**A**) Schematic drawing of the bsAb KN026. (**B**) Thermal stability of KN026 was examined by differential scanning calorimetry (DSC). (**C**) Binding affinity of KN026, trastuzumab (Tmab) and pertuzumab (Pmab) against dual antigens was determined by sandwich ELISA. (**D**) The binding affinity assessment of KN026, trastuzumab (Tmab) and pertuzumab (Pmab) to HER2 on human cancer cells (BT474, NCI-N87, Calu-3).

Based on the heterodimeric Fc variant KiH_ss_-A_k_K_h_, we then constructed a bsAb, termed KN026, targeting two distinct epitopes on HER2 derived from trastuzumab and pertuzumab, which are in clinical usage for the treatment of various cancers (Figure 3A). The bsAb was generated in the format of Fab-Fc heterodimer where the VHCH1 region of trastuzumab was constructed onto the knob chain and that of pertuzumab onto the hole chain (Figure 3A). KN026 were readily expressed in HEK293 cells by transient transfection with three plasmids encoding the two distinct antibody fragments and a common light chain, and purified by protein A affinity column and ionic exchange chromatography.

The thermal stability of KN026 is similar to trastuzumab when examined by differential scanning calorimetry (DSC) with the first transition in the thermogram of KN026 corresponding to CH2 domain unfolding (Tm = 70°C) and the second transition corresponding to CH3 domain unfolding (Tm = 80°C) (Figure 3B). Antigen-specificity of KN026 was determined by sandwich ELISA. A HER2 mutant that can specifically bind to trastuzumab was coated on the plate and the second HER2 mutant (specifically binds to pertuzumab epitope) that probed by biotinylated was added. The results showed that KN026 could bind HER2 antigen concurrently and neither trastuzumab nor pertuzumab could simultaneously bind both HER2 antigens (Figure 3C). To further verify if KN026 binds native HER2 on cell surface, KN026, trastuzumab and pertuzumab were first incubated with HER2-overexpressing cancer cells such as BT474, NCI-N87, Calu-3 and the antibody binding ability was then analyzed using flow cytometry. As shown in Figure 3D, KN026, trastuzumab and pertuzumab could bind to HER2-overexpressing cancer cells dose-dependently with similar EC_50_ values. There are stronger fluorescent intensity from KN026 treated cells (Figure 3D) than those from trastuzumab and pertuzumab treated cells. This indicates that more antibodies bind to HER2 on cell surface due to bi-specificity of KN026 where two KN026 molecules could bind one HER2 molecule on cells surface.

**Figure 4:**
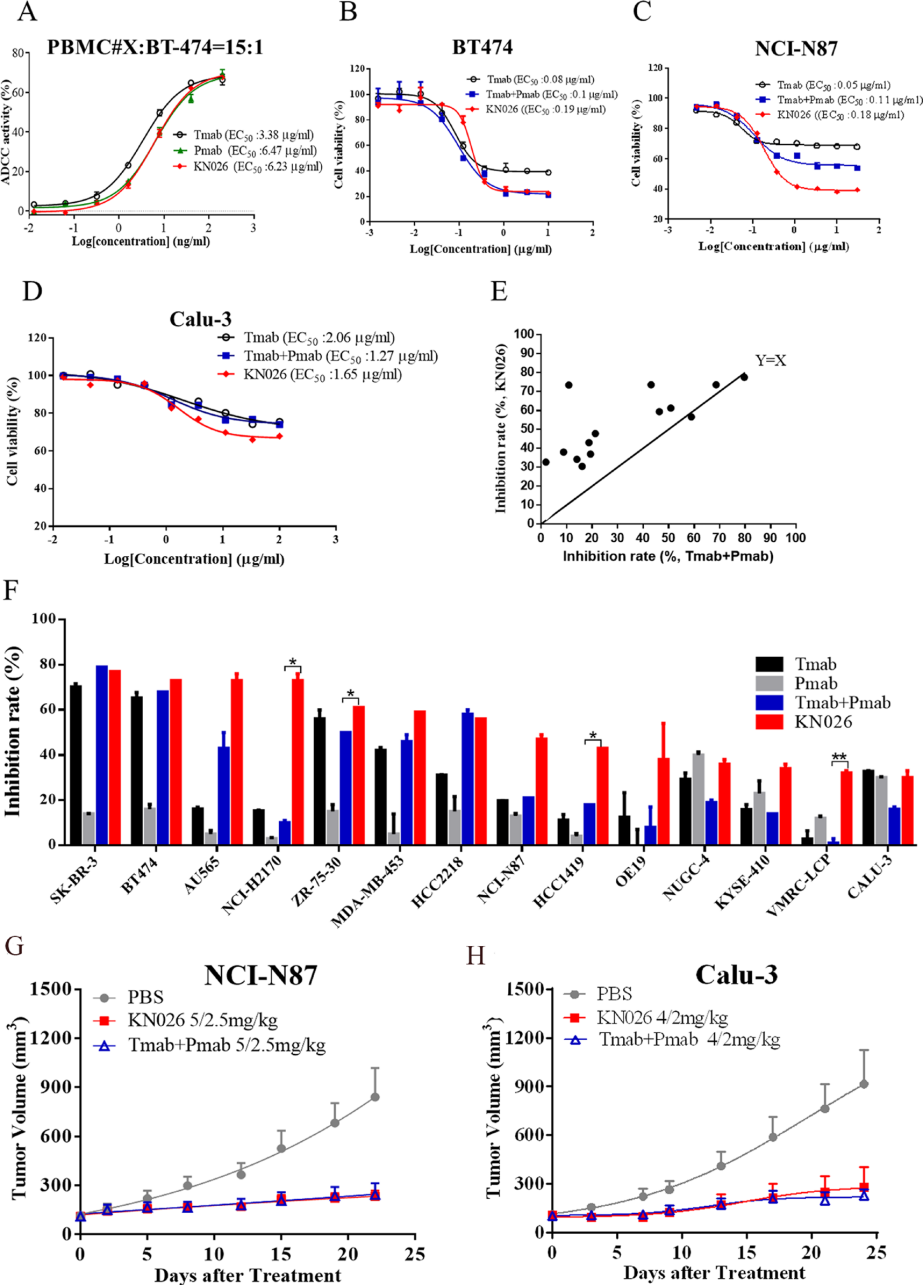
KN026 inhibits proliferation of HER2 over-expressing cancer cells (**A**) The antibody-dependent cellular cytotoxicity (ADCC) of KN026 on HER2-overexpressing cell BT474. (**B**, **C**, **D**) Cell viability by the treatment of KN026, trastuzumab (Tmab), trastuzumab (Tmab) plus pertuzumab (Pmab) at different concentrations on HER2-overexpressing cell BT474, NCI-N87 and Calu-3. Results are shown as percentage of control cell proliferation. (**E**, **F**) The comparison of the inhibitory activity of KN026 and trastuzumab (Tmab) plus pertuzumab (Pmab) on different HER2 expressing cancer cell lines at the concertation of 1 μg/ml (scatter plots: E, column: F). The data above the line indicates superior activity of KN026 in inhibiting cell growth (E). (**G**, **H**) The *in vivo* effect of KN026 and trastuzumab (Tmab) plus pertuzumab (Pmab) in inhibiting tumor growth was assessed on xenograft mice models using HER2-overexpressing gastric cancer cells NCI-N87(G) or NSCLC Calu-3 cells (H). Data are shown as means + SEM.

It has been shown that both trastuzumab [55] and pertuzumab have strong antibody-dependent cellular cytotoxicity (ADCC) activity towards tumor cells [27, 56]. Here we investigated the ADCC activity of KN026 towards BT474 cell using human PBMCs from healthy donor as effector cells. The result showed that KN026 could dose-dependently kill BT474 cells with similar potency to trastuzumab or pertuzumab as single agents (Figure 4A). This indicates that as a dual target bsAb, KN026 retains the strong ADCC activity of its parent molecules.

### KN026 potently inhibits proliferation of HER2 positive cancer cells

We then evaluated the *in vitro* antitumor activity of KN026 using breast cancer cells BT474, gastric cancer cells NCI-N87, and lung cancer cells Calu-3 [26–28]. The cells were incubated with trastuzumab, trastuzumab plus pertuzumab, or KN026 at a series of concentrations and cell viability was assessed after 72 hours (Figure 4). Our result shows that all these cell lines could respond to antibodies treatment but with different sensitivity. Both BT474 (Figure 4B) and NCI-87 cells (Figure 4C) are sensitive to antibodies treatment with EC_50_ around 0.1–0.2 μg/ml while Calu-3 cells are less sensitive with EC_50_ about 2 μg/ml (Figure 4D). Also it appears that KN026 has similar effect on BT474 cells as trastuzumab plus pertuzumab combination while trastuzumab by itself is less effective, which is consistent with previously reported [26]. In treating NCI-87 cells, KN026 is more potent at 1 μg/ml concentration where it kills about 60% of the cells while trastuzumab plus pertuzumab kills about 40% (Figure 4C).

To further assess the efficacy of KN026, we subsequently screened its activity against a panel of 24 HER2 expressing cancer cell lines at different concentrations and compared its activity with that of trastuzumab plus pertuzumab (Supplementary Figure 4). The cell growth inhibition effect of antibodies towards 14 most sensitive cells lines was plotted in Figure 4E and Figure 4F. This result shows that trastuzumab plus pertuzumab has better efficiency than trastuzumab or pertuzumab alone, which is consistent with previous observations [26]. KN026 has similar effect as trastuzumab plus pertuzumab on several cell lines such as SK-BR-3, BT474 etc as expected. Surprisingly, for quite a few cell lines, such as NCI-H2170 and HCC1419, KN026 shows stronger inhibition of cell growth (Figure 4E, 4F). The detailed mechanism underlying this higher efficacy is unclear. We speculate this might be due to some specific properties of these cell lines such as NCI-H2170 or due to the bi-specificity of KN026 in binding two distinct sites of HER2. It is plausible that one KN026 molecule could bind two adjacent HER2 molecules on one cell surface or even linking two cells together, leading to a synergistic effect. In addition, we investigated the *in vivo* antitumor effect of KN026 in HER2-overexpressing NSCLC (Calu-3) and gastric cancer (NCI-N87) tumor cell xenografts. The *in vivo* results shows that KN026 could similarly inhibit tumor growth as trastuzumab and pertuzumab combination (Figure 4G, 4H). Overall these data confirms that KN026 retains the ADCC-mediated mechanism of trastuzumab and pertuzumab, and inhibits proliferation of HER2-overexpressing cancer cells with comparable or better activity as trastuzumab and pertuzumab combination *in vitro* and *in vivo*.

## DISCUSSION

Various bsAbs have been under development in different stages with two approved and many others in clinical trials [53, 57]. So far the knob-into-hole method is one of the most effective ways in generating Fc contained bispecific heterodimer antibodies, however it is not so effective in preventing homodimerization of the hole chain. Here we have addressed this issue by combining knob-in-hole strategy with electrostatic steering method where a complimentary binding surface between Fc chains was generated through F405K and K409A mutations (Figure 1C). Although residue K409 in one CH3 domain forms ionic interaction with D399 of the other CH3 domain in the rim of wild-type Fc interface, removal of this single ionic interaction by K409A mutation is not expected to significantly affect the association of Fc chains. The resulted Fc construct KiH_ss_-A_k_K_h_ confirmed this and it yielded about 94% of Fc heterodimers with total inhibition of homodimer formation of the hole chain (Figure 1D). The crystal structure of this Fc heterodimer solved here reveals an asymmetric, but complimentary interface with lysine side chain of residue 405 occupying the position vacated by K409A substitution. Our molecular modeling confirms that the steric clash and charge repulsion of lysine residue at position 405 would disfavor the homodimer formation.

We further tested the feasibility of KiH_ss_-A_k_K_h_ Fc variant as a platform for the generation of bsAbs. KN026, was generated from trastuzumab and pertuzumab heavy chain and a common light chain. These two anti-HER2 antibodies have distinct epitopes on HER2 [18, 58], and have complementary mechanisms of action (MOA) in in HER2-positive cancer [59, 60]. Here we show KN026 could simultaneously bind two sites in HER2 and retained ADCC activity of trastuzumab. It can also efficiently inhibit proliferation of HER2 expressing cancer cell lines with similar efficacy with the combination of trastuzumab and pertuzumab. Most interestingly, KN026 shows stronger activity in several cell lines such as NCI-2170 than that of trastuzumab plus pertuzumab (Figure 4E, Figure 4F). The detailed molecular mechanism is under further investigation. We also show here that KN026 exhibits similar antitumor activity as trastuzumab plus pertuzumab in NCI-N87 and Calu-3 xenograft mice models. This is consistent with previous bispecific anti-HER2 antibody derived from trastuzumab and pertuzumab, named TP_L_, which potently blocks HER2 heterodimerization and overcomes trastuzumab resistance [61, 62]. Therefore, the strategy of generating a bsAb against different epitopes on the same antigen may provide a promising treatment. Furthermore, from the perspectives of manufacturing process, patient compliance and cost of treatment bsAb may have obvious advantages.

In conclusion, we have engineered a heterodimeric Fc and expounded the structural mechanism underlying its heterodimerization by determining the crystal structure of the Fc variant. Also we shows that a bispecific anti-HER2 antibody KN026 derived from this heterodimeric Fc exhibits comparable antitumor efficacy with the combinations of trastuzumab and pertuzumab antibodies. This demonstrates that the heterodimeric Fc engineered here provides an efficient platform for the generation of bsAbs towards combinational therapy.

## MATERIALS AND METHODS

### Cell lines, antibodies, and animals

All cell lines chosen for screening were accorded with *HER2* copy number amplification (CN > 4) and protein Log2 Expression (> 9) from Cancer Cell Line Encyclopedia (http://www.broadinstitute.org/ccle). The cell lines were purchased from ATCC, JCRB, ECACC and RIKEN, and cultured in appropriate medium plus 10% fetal bovine serum (Invitrogen) at 37°C, 5% CO2 refer to ATCC, JCRB, ECACC and RIKEN. Cell lines were routinely checked for mycoplasma contamination. Trastuzumab was purchased from Roche Ltd. Pertuzumab was produced by Alphamab Co. Ltd. Four- or Five-weeks-old female BALB/c nude mice were obtained from SLACCAS (Shanghai, China). All animals were treated in accordance with guidelines of the Institutional Animal Care and Use Committee.

### Construction, expression and purification

The genes encoding antibodies trastuzumab and pertuzumab were generated by gene synthesis. The Fc/scFv-Fc system was constructed using a scFv derived from trastuzumab. The Fc region contained residues 238-444 of human IgG1. The plasmids encoding two single-chains were cloned to pcDNA4. All substitutions were introduced using KOD Plus Mutagenesis Kit (TYOBO). Positions are numbered according to the EU index. Two paired plasmids with a ratio of 1:1 (scFv-Fc: Fc) were transiently co-transfected to HEK 293H. The proteins were purified from the culture supernatants using Protein A affinity chromatography (GE healthcare). The proteins were identified by reducing and non-reducing SDS-PAGE and quantified by ImageLab (BioRad).

The anti-HER2 bsAb derived from trastuzumab and pertuzumab (named KN026 here), was engineered with Fc region of trastuzumab substituted by knob variant: S354C/T366W/K409A and Fc region of pertuzumab substituted by hole variant: Y349C/T366S/L368A/Y407V/F405K. Two heavy chains and light chains were cloned to pcDNA4 and co-expressed in HEK 293H. Then the secreted antibodies in the expression medium was purified by Protein A affinity chromatography and ion exchange. All antibodies (trastuzumab, pertuzumab and KN026) were dissolved in PBS buffer and stored at −80°C before use.

### Crystallization, data collection and structure determination

The Fc heterodimer fragment (knob-S354C/T366W/K409A, hole-Y349C/T366S /L368A/Y407V/F405K) with an additional 6xhis-tag at the C-terminal of hole chain was purified by Ni column. Crystals of this Fc heterodimer were grown at 22°C under condition of 15% PEG3350, 1 M LiCl, 0.1 M MES pH 6.0 by sitting drop method through mixing 2 μl protein solution (10 mg/ml in 10 mM Tris pH 7.4, 150 mM NaCl) with 2 μl reservoir solution. Crystals appeared after three days. For data collection, single crystal was soaked in 17% PEG3350, 1 M LiCl, 0.1 M MES pH 6.0 and 20% glycerol and rapidly frozen in liquid nitrogen. The diffraction data was collected at SSRF BL17U. Data was indexed and processed with iMosflm and scaled with Aimless from the CCP4 suite [63]. The initial phases were obtained by molecular replacement using Phaser [64] with the structure of human IgG (PDB:3AVE) as the search model. Then structure was refined with Refmac [65], and model building was done with Wincoot [66]. Figures were produced with PyMOL software [67]. The atomic coordinates and structure factors have been deposited in the Protein Data Bank with accession code 5TPS.

### Differential scanning calorimetry

Differential scanning calorimetry (DSC) was performed on a MicroCal VP-Capillary DSC (GE healthcare). Proteins prepared at concentration of 2 mg/ml in PBS, pH 7.4 were heated from 10°C to 110°C at a heating rate of 95°C/hour. The thermogram was fitted using Cp value versus temperature after deduction of the buffer reference scan.

### Sandwich ELISA

ELISA plate was firstly coated by a mutant HER2 protein that only binds trastuzumab (HER2m1) [17, 68] for overnight at 4°C. Then the sample at gradient concentration (1.06–5 μg/ml) was added to the plate, followed by addition of another mutant HER2 protein that only binds pertuzumab (Biotin-HER2m2) [20]. Then the plate was incubated with HRP-streptavidin. The enzymatic reaction was developed by addition of substrate and absorbance was measured.

### Affinity measurement

The affinities of KN026, trastuzumab and pertuzumab for cancer cells were determined by FACS. Briefly, cells were collected and incubated with indicated concentrations of antibodies (KN026, trastuzumab and pertuzumab) for 1 hour. The cells were washed by 1% BSA and were incubated with IgG-FITC (Bioster, China). Then samples were measured using a Flow cytometry (BD).

### Antibody-dependent cell-mediated cytotoxicity (ADCC)

PBMCs were extracted from fresh blood of healthy donator, and maintained in RPMI 1640 with 300 IU/ml IL-2 and 10% FBS for 24 hours. Then the PBMCs were centrifuged and reseeded on 96-well plates with addition of BT474 cells and antibody (KN026, trastuzumab, pertuzumab). After 4–6 hours, the cells were detected with Substrate Solution at 490 nm.

### Cell proliferation assays

The exponentially growing cells were plated into 96-well plates at 1 × 10^4^ cells per well and antibodies at different concentrations were then added after 4 hours. After 6 days treatment, cellular viability was determined using AlamarBlue cell viability assay according to manufacturer's instructions (Life Technologies), and the intensity was measured by SpectraMax M5 plate reader (Molecular Devices, Sunnyvale, CA). Raw values were calculated to evaluate the cell viability or proliferation inhibition rate of these antibodies. Each plate was normalized using the mean value of each condition on the plate vs the mean value of vehicle.

### *In vivo* studies

To initiate tumor xenografts, 4 × 10^6^ cells of human cancer cell lines NCI-N87 or 5 × 10^6^ cells of human cancer cell lines Calu-3 cells were injected s.c. into BALB/c nude mice. When the mean tumor volume was ~100–150 mm^3^, tumor-bearing mice were randomly divided into groups of five or six mice each. Treatments consisted of one or twice weekly intravenous injection of different anti-HER2 antibodies for 3–4 consecutive weeks. Control mice were given PBS alone. Tumors were measured with digital calipers and tumor volumes were calculated by the formula: volume = (length × (width)^2^)/2.

## SUPPLEMENTARY MATERIALS FIGURES AND TABLES


