# Supplementary material for: Structural basis of a novel heterodimeric Fc for bispecific antibody production
Source: Oncotarget. 2017 May 2;8(31):51037–49. doi: 10.18632/oncotarget.17558 (PMC5584228; doi:10.18632/oncotarget.17558)
Supplement: Supplementary file 1 [file oncotarget-08-51037-s001.pdf]

# Structural basis of a novel heterodimeric Fc for bispecific antibody production

## Supplementary Materials

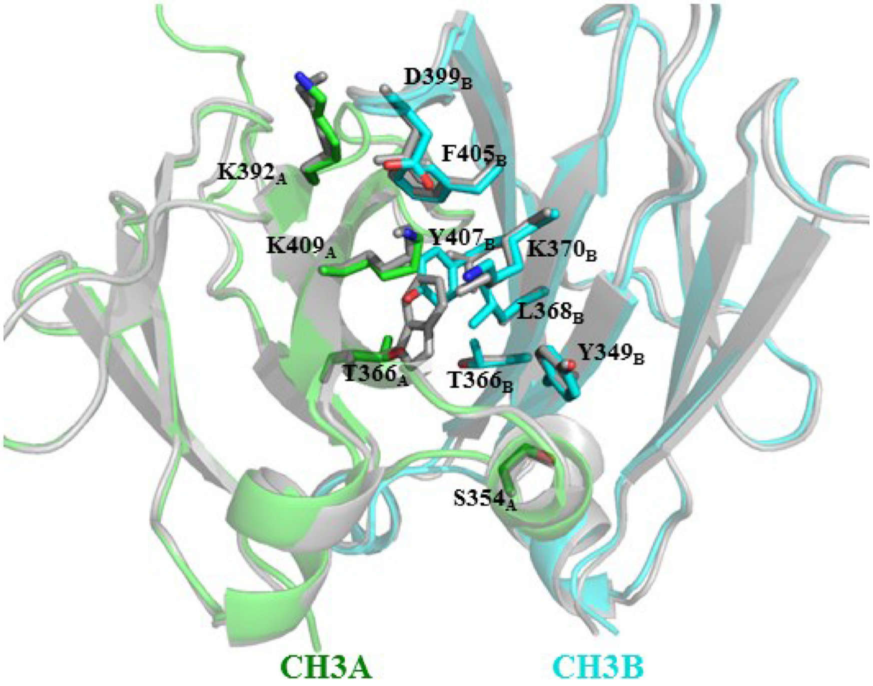


**Supplementary Figure 1: The structure of CH3 domains of wild-type Fc (PDB: 3AVE) is overlaid with the knob-into-hole Fc variant (knob-T366W, hole-T366S/L368A/Y407V, PDB: 4NQS)(gray).** A chain and B chain of wild type are colored by green and cyan, respectively. Key residues of the interface shown in stick.

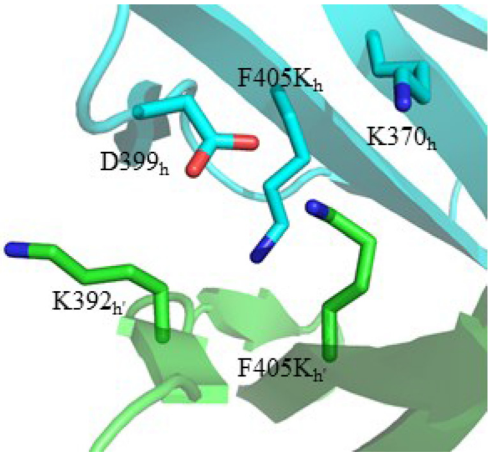


**Supplementary Figure 2: Model of the Structure of hole-hole homodimer of $F405K_h$ and $F405K_h$, indicating unfavorable interactions for its formation.**

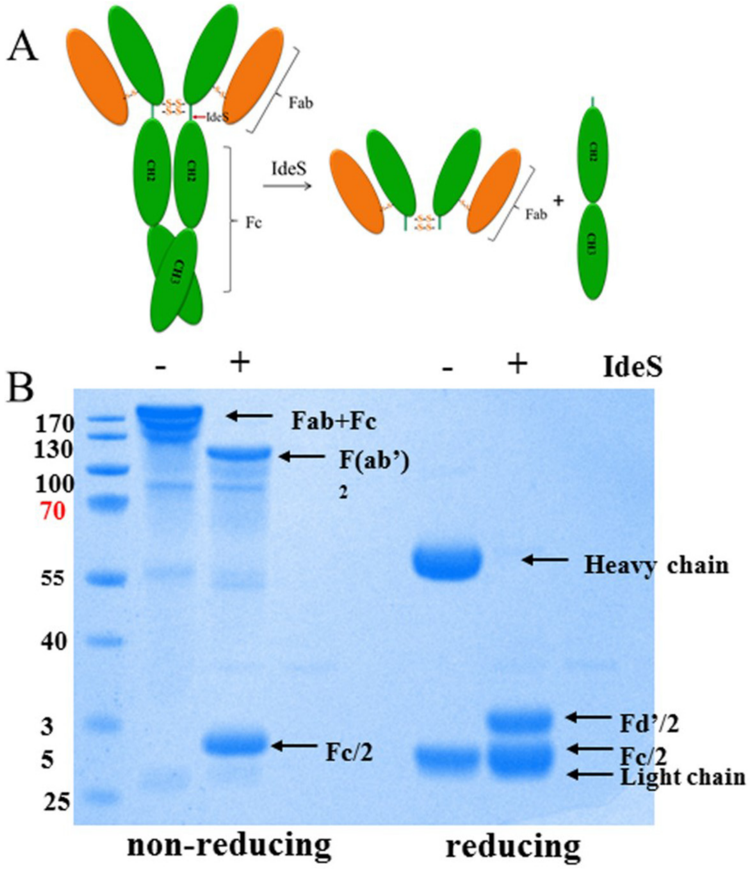


**Supplementary Figure 3: Process of an antibody with wildtype Fc by streptococcal IgG endopeptidase (IdeS).** (**A**) A cartoon shows the antibody products after IdeS treatment. (**B**) SDS-PAGE analysis of normal antibody following IdeS treatment under reducing and non-reducing.

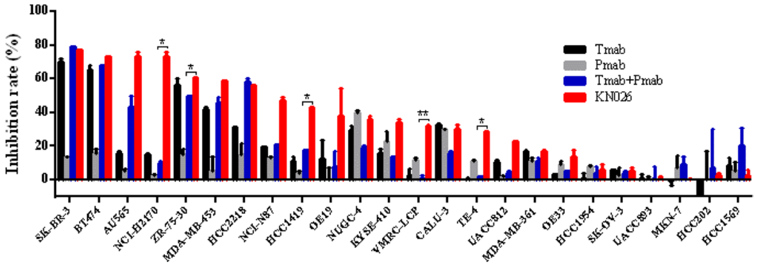


**Supplementary Figure 4: The comparison of the inhibitory activity of KN026 and trastuzumab (Tmab) plus pertuzumab (Pmab) on a panel of 24 HER2 expressing cancer cell lines at the concertation of 1 µg/ml.** Data are shown as means + SEM. *$P < 0.05$, **$P < 0.01$.
